# Pyroptosis: shedding light on the mechanisms and links with cancers

**DOI:** 10.3389/fimmu.2023.1290885

**Published:** 2023-11-02

**Authors:** Hong-mei You, Ling Wang, Hong-wu Meng, Cheng Huang, Guo-ying Fang, Jun Li

**Affiliations:** ^1^ Department of Pharmacy, Hangzhou Women’s Hospital, Hangzhou, China; ^2^ Department of Pharmacy, Shangyu People’s Hospital of Shaoxing, Shaoxing, China; ^3^ Department of Pharmacy, The First Affiliated Hospital of Anhui Medical University, Hefei, China; ^4^ Inflammation and Immune Mediated Diseases Laboratory of Anhui Province, Anhui Institute of Innovative Drugs, School of Pharmacy, Anhui Medical University, Hefei, China

**Keywords:** pyroptosis, gasdermin, caspase, cell death, cancers

## Abstract

Pyroptosis, a novel form of programmed cell death (PCD) discovered after apoptosis and necrosis, is characterized by cell swelling, cytomembrane perforation and lysis, chromatin DNA fragmentation, and the release of intracellular proinflammatory contents, such as Interleukin (IL) 8, IL-1β, ATP, IL-1α, and high mobility group box 1 (HMGB1). Our understanding of pyroptosis has increased over time with an increase in research on the subject: gasdermin-mediated lytic PCD usually, but not always, requires cleavage by caspases. Moreover, new evidence suggests that pyroptosis induction in tumor cells results in a strong inflammatory response and significant cancer regression, which has stimulated great interest among scientists for its potential application in clinical cancer therapy. It’s worth noting that the side effects of chemotherapy and radiotherapy can be triggered by pyroptosis. Thus, the intelligent use of pyroptosis, the double-edged sword for tumors, will enable us to understand the genesis and development of cancers and provide potential methods to develop novel anticancer drugs based on pyroptosis. Hence, in this review, we systematically summarize the molecular mechanisms of pyroptosis and provide the latest available evidence supporting the antitumor properties of pyroptosis, and provide a summary of the various antitumor medicines targeting pyroptosis signaling pathways.

## 1 Introduction

The dynamic balance between cell proliferation and cell death plays a critical role in various physiological and pathological processes in multicellular organisms. Unlike accidental cell death, which is caused by mechanical and physicochemical stimulation, programmed cell death (PCD) can be precisely regulated by genetic and pharmacological factors. Pyroptosis is a recently discovered inflammatory, lytic type of PCD that requires the participation of the membrane-rupturing gasdermin family. Pyroptosis is characterized by cell swelling, membranolysis, and the release of numerous pro-inflammatory factors, including, but not limited to, interleukin (IL)- 1β, IL-18, ATP, and high mobility group box 1 (HMGB1) (1–4). As the release of numerous inflammatory cytokines outside the cells triggers inflammatory reactions, pyroptosis is also called inflammatory “necrosis” (5, 6).

In1992, scientists first discovered pyroptosis phenomenon via experiments involving electron microscopy in mouse macrophages and human monocytes infected with *Shigella flexneri* or *Salmonella*. Whereas, it was incorrectly identified as apoptosis due to the limitations of technology (7). As the cell death caused by *Salmonella* could be blocked after knocking out caspase-1 (8), the process was mistakenly deemed to caspase-dependent apoptosis originally. However, in 2001, the laboratory of Cookson revealed that the macrophage death induced by *Salmonella* presented completely different characteristics to that of apoptosis, and the term “pyroptosis” was first proposed from the Greek roots *pyro*, relevant to fever or fire, and *ptosis* (9). Researchers at that time still considered “pyroptosis” as caspase-1-dependent programmed necrosis. However, with the discovery and identification of the new protein, gasdermin D (GSDMD) in 2015, the underlying mechanisms of pyroptosis have been gradually revealed (10). It was discovered that activated caspases specifically cleaved GSDMD and then released the amino-terminal gasdermin-N (GSDMD-NT), which resulted in pore formation in the cell membrane, leading to cell swelling, and osmotic lysis. This study demonstrated that GSDMD is a critical and central executor of pyroptosis (10). In addition to GSDMD, there are five other members of the gasdermin family. The human gasdermin family includes GSDMA, GSDMB, GSDMC, GSDME/DFNA5, and PVJK/DFNB59. Moreover, the mouse gasdemin family includes GSDMA, GSDMC, GSDMD, GSDME, and PJVK/DFNB59 (11, 12). Members of this family play various roles in different biological processes. Other than DFNB59, the inactive gasdemins consist of an active N-terminal domain and a suppressive C-terminal domain linked by a pliable peptide (13). The pore-forming ability of the N-terminus is inhibited by binding to the C-terminus (14). In 2017, Wang et al. discovered that cisplatin, decitabine, and other classical chemotherapy medicines could induce pyroptosis via caspase-3-induced cleavage of GSDME (15). Thus, Feng Shao et al. redefined the concept of pyroptosis as the gasdermin family-mediated regulated necrotic cell death (16). In 2018, the Nomenclature Committee on Cell Death proposed to redefine pyroptosis as a type of regulated cell death that critically relies on the formation of pores in the plasma membrane caused by members of the gasdermin family, often (but not always) as a consequence of inflammatory caspase activation (17).

According to the World Health Organization, cancer is the leading cause of death among the elderly populations globally. Non-communicable diseases, including cancer, account for 71% all deaths annually (18–20). Despite pharmaceutical and technological advancements in cancer treatment, the morbidity and mortality rates of cancer have stayed relatively stable (21). In 2020, 18% of cancer-associated deaths were due to lung cancer. Furthermore, lung, colon, stomach, female breast, esophageal, and liver cancers collectively accounted for approximately 50% of cancer mortality in the same year (20). The growing number of cancer cases and consistently high mortality of tumors stress the necessity and urgency to explore further mechanisms and develop more efficient targets to improve patient’s quality of life and cancer prognosis.

Many studies have shown a significant relationship between pyroptosis and a variety of human malignant tumors. Pyroptosis appears to play a double-edged role in the development and progression of cancer. Generally speaking, activation of pyroptosis pathways is associated with tumor-suppressive immunity. However, various signaling pathways and the excessive release of inflammatory factors during pyroptosis are also associated with tumorigenesis and resistance to multiple chemotherapeutic drugs (22, 23). What’s more, pyroptosis suppression can accelerate tumor growth and metastasis (22, 24). Recently, numerous studies have explored the molecular mechanisms and potential treatment strategies for human cancers via targeting pyrotosis (25–27).

In this review, we provide a comprehensive overview of the latest developments in exploring the molecular mechanisms of pyroptosis and the relationship between pyroptosis and various human tumors. Furthermore, we demonstrated the possibility of applying drugs to treat tumors by targeting pyroptosis-associated genes.

## 2 Mechanisms regulating pyroptosis

As described above, pyroptosis can result in the release of various cytokines, accelerate T lymphocytes and macrophages activation, induce an intense inflammatory response, and contribute to immune phagocytosis (28, 29). Numerous human diseases are associated with mutations in inflammasome complexes (30). Thus, an in-depth exploration and comprehensive summary of the mechanisms of pyroptosis may provide novel, and promising directions for the clinical treatment of human diseases.

### 2.1 The caspase-1-induced canonical inflammasome pathway

**Figure 1:**
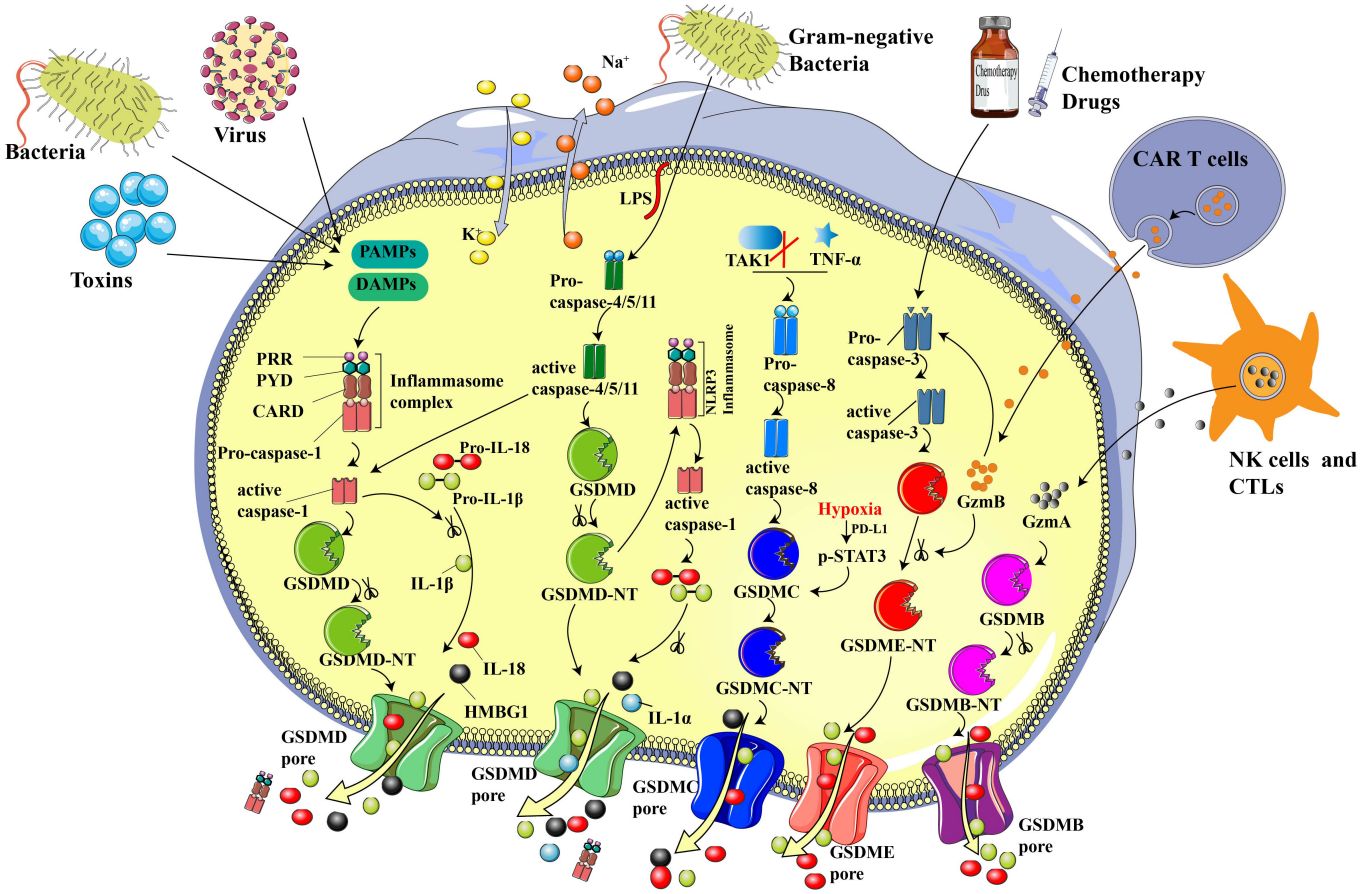
Sketch of essential components and primary mechanisms of pyroptosis. In the canonical inflammasome pathway, PRRs recognize DAMP and/or PAMP, leading to the assembling and activation of inflammasomes and subsequently contributing to the recruitment and activation of pro-caspase-1. GSDMD and pro-IL-18/1β will be cleaved to N-GSDMD and inflammatory IL-18/1β by acitivated cleaved-caspase-1. N-GSDMD punches noneselective pores on cell membrane, resulting in water influx, cell swelling, inflammatory factors release, and cell death. In the non-canonical pathway, Gram-negative bacteria-derived LPS cleaves and activates caspase-4/5/11, inducing pyroptosis via cleaving GSDMD. Chemotherapy drugs can trigger pyroptosis through stimulating caspase-3 and GSDME cleavage. N-GSDME possesses the similar cell membrane perforation function to N-GSDMD, which leads to cellular perfusion, increased intracellular osmotic pressure and various inflammatory mediator outflow. In addition, CTLs- and NK cells-released GZMA can cleave GSDMB to N-GSDMB, contributing to the GSDMB pore formation on cell membrane and induce pyroptosis. CAR-T cells-derived GZMB can cleave GSDME to N-GSDME and induce pyroptosis. What’s more, TAK1 inhibition and TNF-α are able to activate caspase-8, which will further cleave GSDMC to N-GSDMC and induce pyroptosis. Under hypoxia, the transcription and cleavage of GSDMC specifically mediated by caspase-8 is enhanced via p-STAT3 physically interaction with PD-L1.

According to the canonical theory, pyroptosis primarily depends on inflammasomes to activate the caspase family and generate a variety of pathophysiological reactions (31, 32). Inflammasomes are multi-molecular complexes that is comprised of pattern recognition receptors (PRRs - the sensor proteins), apoptosis-associated speck-like proteins (ASC - the adaptor protein), which contain a caspase-recruitment domain, and inflammatory caspases (16, 22, 33). The most prevalent PRRs include nucleotide-binding oligomerization domain-like receptors (NLRs), namely NLRP1, NLRP3, and NLRP4, absent in melanoma 2 (AIM2), and pyrin (34, 35). Danger-associated molecular patterns (DAMPs, e.g., heat shock proteins, fibrinogen, and DNA) and pathogen-associated molecular patterns (PAMPs; e.g., glycans, lipopolysaccharides, and flagellin) can be precisely recognized by various forms of PRRs (30, 36). What’s more, ASC is composed of a pyrin domain (PYD) and a caspase activation and recruitment domain (CARD), which is essential for the recruitment and activation of pro-caspase-1 (37). Scientists have reported that upon stimulation of PRRs, the inflammatory caspase family can activate GSDMD via cleavage at the Asp275 (mouse Asp 276) site to form the 31 kDa N-terminus (N-GSDMD) and 22 kDa C-terminus (C-GSDMD), then the N-GSDMD punches holes in the cell membrane, resulting in inflammatory factors release and cell pyroptosis (6, 38). Besides, IL-1β and IL-18 can also be cleaved by caspase-1 to become mature IL-1β and IL-18, which will be secreted through the holes produced by N-GSDMD (39) (**Figure 1**).

Notably, pyroptosis exihibits a dual role in its impact on human health. On the one hand, the pyroptotic death of tumor cells leads to cell swelling and rupture, thereby suppressing the occurrence and development of tumors. However, on the other hand, pyroptosis of non-cancerous cells caused by chemotherapeutic drugs damages normal organs, which is probably one reason for the toxic side effects caused by chemotherapeutic drugs (15). Furthermore, the released inflammatory factors, such as IL-1 and IL-18, caused by pyroptosis, form a chronic inflammatory microenvironment, which will accelerate the genesis and development of tumors (4, 40). Thus, it is necessary and prudent to develop novel pyroptosis inducers that can specifically target tumor cells without damaging normal cells.

Recently, Ge et al. developed a copperbacteriochlorin nanosheet (Cu-TBB), a tumor-specific inducer of pyroptosis. Interestingly, Cu-TBB can remain in a deactivated state in normal cells and organs, it can switch to an activated state in tumor microenvironment with high GSH levels, contributing to Cu^+^ and TBB release (41). The released Cu^+^ and TBB subsequently trigger cascade reactions to generate multiple ROS species, including O_2_-•, ^1^O_2_, and virulent ·OH in cells. Mass production of ROS can activate caspase-1/GSDMD-mediated pyroptosis and subsequently promote numerous inflammatory cytokines, enhancing dendritic cell maturation, and T lymphocyte initiation, thereby concurrently accelerating primary tumor elimination and suppressing the growth and metastasis of distant tumors (41).

### 2.2 The non-canonical pathway

Different from the canonical inflammasome pathway, the non-canonical pathway does not rely on caspase-1. Instead, it depends on caspase-11 in mice and caspase-4/5 in humans (42). Activation of these caspases can be induced via the direct binding of intracellular lipopolysaccharide (LPS) by the N-terminal CARD (43). As shown in **Figure 1**, LPS originates from gram-negative bacteria, which may be delivered to the cytoplasmic via infection and membrane vesicles (44). After caspases activation, GSDMD will be cleaved into N-GSDMD by activated caspase-4/5/11 at the same site as caspase-1. The liberated N-GSDMD then alters conformation to oligomerize and form irreparable transmembrane β-barrel holes, ultimately causing the efflux of K^+^ (45, 46). Activated caspase-11 can induce pyroptosis directly and can also activate capsase-1 via cleaving GSDMD (10). Though caspase-4/5/11 are not able to split pro-IL-1β/18, they can participate in the process of maturation and release of IL-1β/18 via the NLRP3/caspase-1 pathway, which is accompanied by the secretion of high mobility group box1 (HMGB1) and IL-1α in the meantime (16).

### 2.3 The caspase-3 and GSDME-mediated pyroptosis pathway

In-depth studies are gradually uncovering the mechanisms and functions of GSDM family proteins. GSDME was originally recognized as one mutated gene (DFNA5), which leads to hearing disorders, contributed by loss of cochlear hair loss (47–49). In 2017, scientists reported that chemotherapy could switch cell death from apoptosis to pyroptosis via caspase-3-induced GSDME cleavage in tumor cells with high GSDME expression (15, 50). The generated N-GSDME will then transfer to the cell membrane and punch pores for pyroptosis induction. It is worth noting that GSDME is expressed at low levels in various tumors but is highly-expressed in many normal organs. GSDME-dependent pyroptosis occurs in various primary human cells after caspase-3 activation by chemotherapeutic drugs (15). Therefore, GSDME-mediated pyroptosis may be one important cause of the severe side effects of cancer chemotherapy.

Notably, numerous studies have demonstrated that GSDME exhibits low or absent expression levels in most tumor cells because of promoter hypermethylation. In contrast, it is highly expressed in normal tissues (4, 15, 51, 52). Furthermore, cells with high GSDME content undergo pyroptosis following the “apoptotic stimulation,” such as that provoked by chemotherapeutic drugs (15, 50). An in-depth investigation revealed that caspase-3/GSDME-induced pyroptosis in normal cells and organs results in the toxic side effects of chemotherapeutic drugs. These findings revolutionized our incomplete understanding of the anti-tumor effects of pyroptosis in tumor progression. This highlights the importance of developing new compounds or materials specially targeting tumor cell pyroptosis while preserving normal cells. In addition, targeting GSDME in normal cells may be an effective approach to attenuating chemotherapy-induced toxicity.

### 2.4 The granzyme-mediated pyroptosis pathway

Cytotoxic T lymphocytes (CTLs) and natural killer cells (NKs) are identified as the key effectors that target and kill transformed and virus-infected cells, respectively. Scientists previously thought that the immune ability of cytotoxic lymphocytes depended on granzyme-induced targeted cell apoptosis. Until 2020, Shao et al. (53) discovered that pyroptosis is also one of the mechanisms by which CTLs and NKs target GSDMB-positive cells. As shown in **Figure 1**, lymphocytes-derived granzyme A (GZMA) cleaves GSDMB to N-GSDMB at the Lys229/Lys244 site, causing pore formation, cellular swelling, cell rupture, and a series of pyroptotic phenomena. Additionally, interferon-γ was proven to up-regulate the expression of GSDMB and enhance pyroptosis (53). Liu et al. reported that CAR T cells promptly activate caspase-3 in B leukemic cells and other target cells via the release of granzyme B (GZMB), which further activates the caspase-3/GSDME-participating pyroptotic pathway and contributes to extensive pyroptosis. As a result, pyroptosis-freed inflammatory factors will further activate caspase-1/GSDMD and the MAPK-NF-κB pathway, causing cytokine release syndrome (CRS) (54). The above studies confirmed the essential role of the GZMA/GSDMB and GZMB/GSDME pathways in anti-tumor immune processes, providing a novel approach for cancer immunotherapy.

## 3 Links between pyroptosis and human cancers

**Figure 2:**
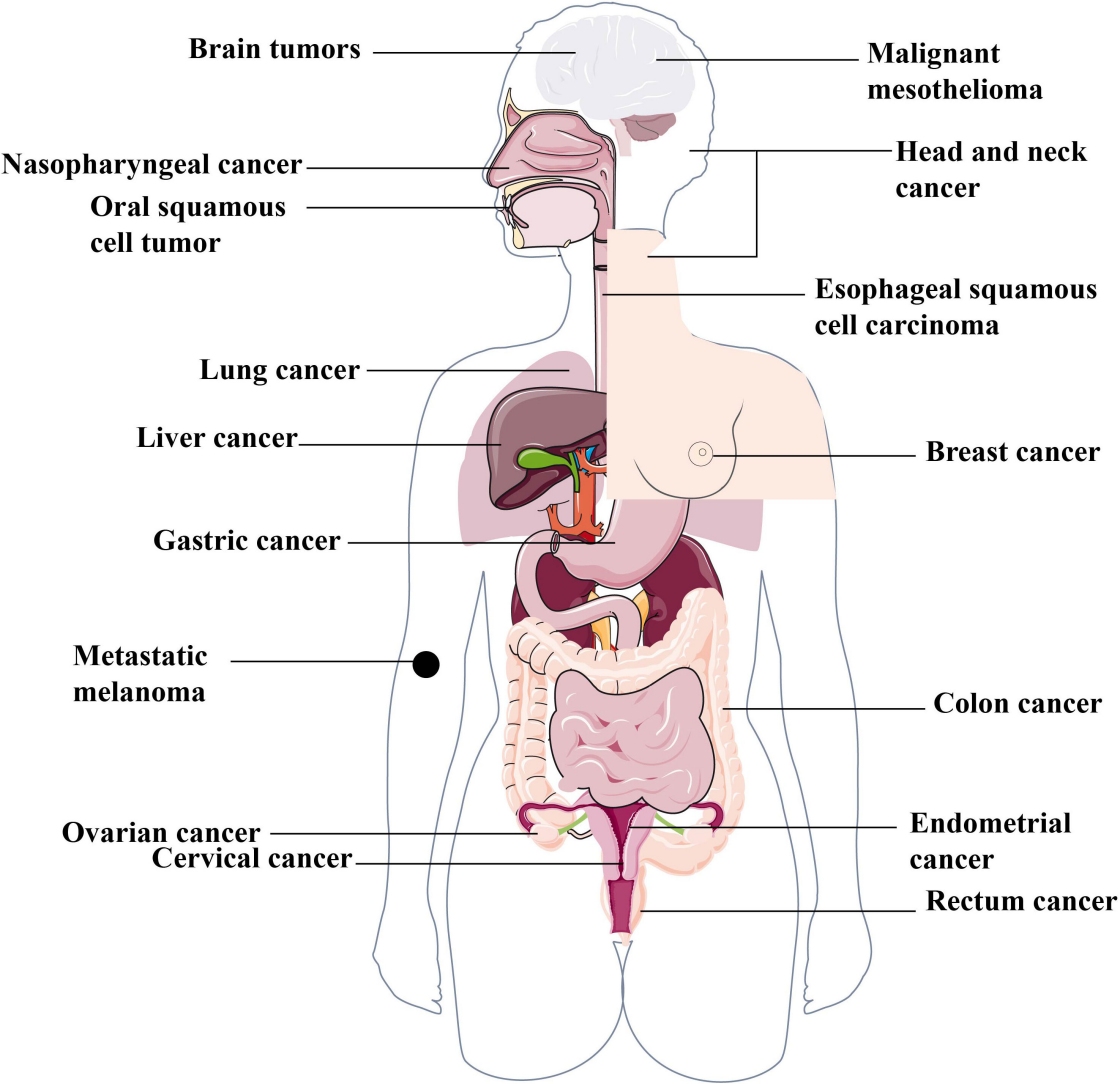
Various human cancers associated with pyroptosis. The complex function of pyroptosis is widely involved in different human cancers, including but not limited to the reproductive system cancers, such as breast cancer, ovarian cancer, cervical cancer etc. the digestive system cancers, such as gastric cancer, liver cancer, colorectal cancer, oral squamous cell carcinoma, esophageal squamous cell carcinoma etc. the respiratory system cancer, such as lung cancer, nasopharynged carcinoma etc. the nervous system cancer, such as brain tumors and malignant mesothelioma etc.

Many studies have demonstrated that pyroptosis plays an important role in tumor progression and anti-tumor immunity (**Figure 2**) by serving as a double-edged sword with tumor-promoting and inhibitory abilities. On the one hand, brachychronic and excessive pyroptosis may lead to plentiful infiltration of immune cells, inducing numerous tumor cell death and activation of anti-tumor immunity to suppress tumor development (1). On the other hand, long-term chronic pyroptosis induces pro-inflammatory factors that accelerate the formation of an inflammatory microenvironment, which is suitable for tumor growth (55). In addition, certain side effects of chemotherapy and radiotherapy may also be triggered by pyroptosis. Thus, a comprehensive understanding of the relationship between pyroptosis and human cancers may provide us with the potential and novel treatment strategies for a wide spectrum of human tumors in the clinic. This following section of this review will summarize the current research progress into the potential application of pyroptosis-targeted therapies for various human tumors.

### 3.1 Pyroptosis and hepatocellular carcinoma

Hepatocellular carcinoma (HCC) is the main histological subtype of human primary liver cancer globally (56). In 2013, Wei et al. reported that the expressionof NLRP3 inflammasome components was either significantly downregulated or completely absent in HCC. Moreover, this downregulation was closely associated with tumor progression and poor pathological differentiation (57). In further studies, they further demonstrated that the ability of 17β-estradiol to suppress HCC was through 17β-estradiol-induced the NLRP3 inflammasome activation, which trrigered pyroptotic cell death (58). Sorafenib, a kind of kinase inhibitor, is approved for treatment of HCC. Hage et al. revealed that sorafenib-treated MΦ presented caspase-1-activated pyroptosis, contributing to the release of numerous proinflammatory cytokines and the proliferation of NK cells, ultimately suppressing the development and metastasis of tumor cells (59). Additionally, miltirone was proven to possess anti-HCC activity via the caspase-3/GSDME-mediated pyroptosis pathway (60). Mechanistically, miltirone effectively inhibited reactive oxygen species accumulation and restrained mitogen-activated and extracellular signal-regulated kinase (MEK) phosphorylation, which inhibited extracellular regulated protein kinase 1/2 (ERK1/2) activity, and ultimately triggering pyroptosis in tumor cells (60). In addition, abnormally down-regulated expression of caspase-1, IL-18, and IL-1β expression was observed in HCC tissues compared with these in the adjacent normal tissues (61, 62).

### 3.2 Pyroptosis and lung cancer

In previous studies, various pyroptosis pathway members, including but not limited to inflammasomes, gasdermins, and various inflammatory cytokines, have been reported to participate in the genesis, proliferation, metastasis, and invasion of pulmonary cancer (63–65). Depending on the specific contexts, these components play diverse and even sometimes opposing roles in tumor promotion and therapeutic processes. In 2015, Wang et al. designed a study to investigate the role of the NLRP3 inflammasome in the proliferation and migration of the A549 lung cancer cell line and observed that activation of the NLRP3 inflammasome via LPS+ATP promoted the above process. Besides, increased expression of phosphorylation of Akt, ERK1/2, and Snail, decreased E-cadherin expression phenomenon was observed after the NLRP3 inflammasome activation (65). Taken together, these results manifested that NLRP3 inflammasome activation promotes the proliferation and migration of A549 cells via regulating downstream proteins. Later, the NLRP3 inflammasome was further demonstrated to be associated with the malignant transformation of BEAS-2B bronchial epithelial cells (63). In addition to the NLRP3 inflammasome, the AIM2 inflammasome was reported to present high expression levels in non-small cell lung cancer cells (NSCLCs) and to exert tumor-promoting ability both *in vivo* and *in vitro* (66, 67). Interestingly, higher GSDMD expression was related to more severe carcinogenesis with a larger tumor size and more progressive tumor metastasis, which was indicative of a poor prognosis for lung adenocarcinoma (68). Unlike the above studies, scientists gradually discovered the suppressive role of pyroptosis-associated proteins in the progression of lung cancer. Sakaizawa et al. reported that knockdown of ASC, an essential element of inflammasomes, may accelerate the proliferation, motility, and invasion of A549 cells via increasing the expression of Bcl-2 and phosphor-Src. What’s more, ASC-knockdown cells showed resistance ability to cisplatin. Thus, the above study indicated that ASC may play a role in the inhibition of lung cancer via inhibiting Bcl-2 and p-Src (64). Similarly, it has been shown that high expression of GSDME can improve the sensitivity of lung cancer cells to drugs. In contrast, loss of GSDME promotes drug resistance (69).

### 3.3 Pyroptosis and gastric cancer

Gastric cancer is a common gastrointestinal tumor that ranks fifth in morbidity and fourth in mortality among all malignant tumors worldwide (20). As the important elements in pyroptosis, gasdermin family proteins have been shown to participate in the initiation and development process of gastric cancer. Studies have confirmed that GSDMD expression is significantly decreased in gastric cancer cells compared to that in normal cells, resulting in the proliferation of cancer cells. An in-depth mechanistic study showed that the content of cyclin A2 and cyclin-dependent kinase were decreased by high expression of GSDMD via ERK1/2, STAT3, and PI3K/AKT inhibition, suppressing S/G conversion in GC cells and exerting the anti-GC capacity of GSDMD (70–72).

Additionally, recent studies have indicated that GSDME, rather than GSDMD, can switch chemotherapy-induced caspase-3-dependent cell apoptosis to pyroptotic cell death in gastric cancer cells (15, 27). Furthermore, GSDMA and GSDMC levels are decreased in GC and are regarded as potent cancer suppressor genes (73–75). Komiyama et al. discovered that GSDMB was not detected or displayed deficient expression levels in normal gastric tissue samples. Conversely, the pre-cancer and cancerous samples show moderate or augmented GSDMB expression, suggesting that the rich content of GSDMB may be related to tumor invasion (76). It’s worth noting that overexpression of GSDME in gastric cancer cells slowed down cell proliferation compared to that in control-group cells (52), suggesting that GSDME may play a role in tumor suppression. In addition, the expression of GSDME was shown to be upregulated in tumor cells after treatment with decitabine, contributing to pyroptosis and promoting cancer cells’ sensitivity to chemotherapy agents (12, 15).

### 3.4 Pyroptosis and breast cancer

Breast cancer has become the most common malignant tumor among women. In China, 400,000 breast cancer cases are reported annually, accounting for 16.5% of malignant cancers in women (20). Numerous studies have indicated that pyroptosis plays an important role in breast cancer progression. Jiao et al. demonstrated that human umbilical cord mesenchymal stem cells (hUCMSCs) could be used as a novel tool for tumor therapy. They found that the conditional medium treated with factors secreted from hUCMSCs could cause pyroptosis in breast cancer cell line MCF7, presenting a potential cell-based breast tumor therapy (77). Additionally, docosahexaenoic acid (DHA) has been proven to trigger MDA-MB-231 breast cancer cell pyroptosis via the caspase-1/GSDMD pathway, inducing IL-1β secretion, translocation of HMGB1 to the cytoplasm, and pore formation in the cell membrane (78). Surprisingly, more and more compounds targeting pyroptosis have been explored for treating breast cancer. For instance, Li et al. established breast cancer xenograft models and utilized several methods to analyze inflammasome levels and cell proliferation. The results showed that cells and mice treated with dihydroartemisinin had higher AIM2, caspase-3, and GSDME levels, indicating proliferation inhibition and pyroptosis activation (79). As a typical anthracycline antitumor drug, doxorubicin can induce ROS accumulation, JNK phosphorylation and ultimately promote pyroptosis through the caspase-3/GSDME pathway in breast cancer cells. However, GSDME is also expressed in cardiomyocytes, whose pyroptosis is one of the major causes of doxorubicin-induced cardiovascular side toxicity (80). Thus, successful clinical therapeutic strategies will require the proper, informed use of pyroptosis-targeted agents.

### 3.5 Pyroptosis and brain tumours

Although rare, brain and other central nervous system tumors contribute to severe morbidity and mortality and are the most common solid tumors in pediatric patients (81). Among them, glioblastoma multiforme (GBM) is classified as a grade IV glioma by the World Health Organization and is the most prevalent and devastating primary tumor (82). Utill now, adjuvant radiation therapy after surgical resection and oral temozolomide chemotherapy have been the most common and effective therapeutic strategies for patients with GBM. Whereas, temozolomide, the unique clinical medicine available for GBM, often causes frequent toxic side effects and drug resistance (83, 84). Thus, there is an urgent need to develop novel targeted compounds with higher safety and fewer side effects to treat brain tumors in order to meet clinical needs.

Li et al. (85) discovered that galangin, a natural flavonoid, possesses antitumor ability via inducing, pyroptosis, apoptosis, and autophagy, leading to the suppression of GBM growth *in vivo* and *in vitro*. What’s more, a study by Du et al. (86) demonstrated that benzimidazoles had potent activity of averting GBM by inducing GBM cell cycle arrest at the G2/M stage through the P53/P21/cyclin B1 signaling pathway, and simultaneously inducing chondriosome-dependent apoptosis and triggering GBM cell pyroptosis via the NF-κB/NLRP3/GSDMD signaling pathway *in vivo* and *in vitro*. Thus, benzimidazoles may be promising candidates for the treatment of TMZ-resistant or TMZ-less active patients through blocking the cell cycle and promoting cell apoptosis and pyroptosis concurrently. Additionally, several studies have demonstrated that GBM cell lines treated with 4, 5-Dimethoxycanthin-6-one (87) or kaempferol (88) undergo cellular-swelling pyroptosis and exhibit anti-GBM activity. In addition to these compounds, certain noncoding RNAs have been reported to inhibit GBM through inducing pyroptosis via regulating multiple molecular pathways. Meng et al. (89) revealed that the knockdown of has-circ-0001836 markedly suppressed the viability, induced pyroptosis of glioma cells, and restrained tumor growth via epigenetically elevating NLRP1 content. In addition, several studies have demonstrated the inhibitory effect of miR-214 on the proliferation and migration of GBM cell lines through caspase-1-induced pyroptosis in gliomas (26, 90, 91). To date, a growing number of studies have focused on brain tumors and the development of novel targets and drugs targeting pyroptosis would accelerate the progression of brain tumor treatment.

### 3.6 Pyroptosis and colorectal cancer

Colorectal cancer (CRC) is the second leading cause of cancer-associated deaths and the third most common malignancy in the world (92). Because of the poor prognosis of CRC and the burden on patients’ daily lives and their mental and financial well-being, there is an urgent need to explore effective therapeutic strategies to improve the prognosis and patients’ quality of life. Numerous studies have revealed a strong relationship between pyroptosis and CRC, and pyroptosis has been considered as an essential target for CRC treatment. Li et al. demonstrated that secoisolariciresinol diglucoside (SDG) exerts its anti-CRC properties through inducing GSDMD-dependent pyroptosis through activation of the ROS/P13K/AKT/BAK-mitochondrial apoptosis pathway (93). What’s more, Mangues et al. developed nanostructured toxins, which were able to alternatively eliminate drug-resistant CXCR4^+^ CRC cancer stem cells through triggering GSDMD-mediated pyroptosis (94). Notably, the development of compounds targeting GSDME-dependent pyroptosis may be a promising approach for effectively preventing metastatic CRC tumors. In the colitis-associated colorectal cancer model, the GSDME^-/-^ mice showed colon shortening, reduced weight loss, and decreased tumor size compared to their WT littermates. Further treatment with anti-HMGB1 antibodies decreased tumor size, ERK1/2 activation, and PCNA expression in azoxymethane/dextran sodium sulfate-treated WT mice, implying that GSDME-associated pyroptosis accelerates colitis-associated colorectal cancer progression via intracellular HMGB1 release, triggering cancer cell proliferation (95). In addition, several other compounds, such as A438079 (96) and GW4064 (97), were also demonstrated to regulate CRC development via targeting pyroptosis. These studies may provide a research basis for future development of promising drugs to improve the therapeutic effects of CRC.

### 3.7 Pyroptosis and other cancers

In addition to the cancers mentioned above, pyroptosis plays various roles in numerous other types of cancers. Yu et al. reported that lobaplatin-induced pyroptosis is mediated by the GSDME/ROS/JNK/Bax-mitochondrial apoptotic signaling pathway and that further activation of caspase-3/9 is a novel mechanism by which lobaplatin eradicates colon cancer cells (98). Additionally, So et al. discovered that the absence of SIRT1 could enhance AIM2 inflammasome-mediated pyroptotic death in cervical cancer cells, which might be a potential target for the effective treatment of cervical cancer (99). Malignant mesothelioma (MM), a pleural or periotoneal mesothelial cellular cancer caused by asbestos is particularly resistant to chemoradiotherapy (100). Pyroptosis is suppressed in MM tumor cells judged from the low expression of NLRP3 and caspase-1. Treatment of MM cells with doxorubicin or cisplatin causes the activation of NLRP3/caspase-1 and excessive secretion of pro-inflammatory factors, resulting in pyroptosis of MM cells (101). Accumulating evidence have also manifested that miRNAs and lncRNAs are closely associated with pyroptosis in the generation and metastasis of specific tumor. For example, Liu et al. demonstrated that the overexpression of lncRNA-XIST promoted the development of NSCLCs via sponging miR-335, leading to increased SOD2 levels, reduced ROS generation, and NLRP3 inflammasome activation. In other words, the absence of lncRNA-XIST suppressed the NSCLCs progression via miR-335/SOD2/ROS pathway-mediated pyroptosis (102). Furthermore, miR-214 plays an important role in restraining the proliferation and migration of glioma cells through targeting caspase-1-involved pyroptosis (26). Another recent study discovered that lnc-NEAT1 accelerates ionizing radiation-caused pyroptosis through the miR-448/GSDME pathway in colorectal cancer cells (103). An increasing number of novel mechanisms of pyroptosis have been explored, providing more and more promising possibilities for targeted cancer therapy.

## 4 Compounds targeting pyroptosis

As shown in **Table 1**, many basic and clinical trials have demonstrated that pyroptotic signaling pathways are closely related to tumor genesis and development. An increasing number of compounds that directly or indirectly target pyroptosis-associated molecules have been shown to play important roles in tumor suppression via contributing to or enhancing pyroptosis. It’s worth noting that some of these compounds, such as cisplatin, metformin, and doxorubicin, have been approved by the Food and Drug Administration and applied in clinical treatment for years. However, research on their mechanisms through pyroptosis has been lacking. In this review, we have come up with a list of compounds that target pyroptosis, which may provide novel ideas for clinical treatment of cancer.

**Table 1:** Compounds targeting pyroptosis in various cancers.

Compounds	Targets	Function	Mechanisms	Cancers	References
Cisplatin	Caspase-1/GSDMD	Suppressing tumor growth and metastasis	Upregulating lncRNA MEG3 and activating NLRP3/caspase-1/GSDMD pathway, accelerating IL-18/IL-1β release.	Triple-negative breast cancer	(104)
Cucurbitacin B	Caspase-1/GSDMD	Suppressing tumor progression	Binding with TLR4 and activating NLRP3/caspase-1/GSDMD pathway, inducing cell swelling and HMGB1/IL-18/IL-1β release.	Non-small cell lung cancer	(105)
Cu-TBB	Caspase-1/GSDMD	Suppressing tumor progression	Promoting the accumulation of ROS and activating caspase-1/GSDMD mediated pyroptosis, inducing inflammatory factors release.	Cervical cancer and liver cancer	(41)
Anthocyanin	Caspase-1/GSDMD	Restraining tumor cell viability and suppressing proliferation and migration ability.	Activating NLRP3/caspase-1 and degrading GSDMD, releasing inflammatory factors.	Oral squamous cell tumor	(106)
PPVI	Caspase-1/GSDMD	Inhibiting tumor cell proliferation.	Activating NLRP3/caspase-1 and degrading GSDMD, releasing inflammatory factors.	Non-small cell lung cancer	(107)
DHA	Caspase-1/GSDMD	Exerting anticancer effects.	Activating caspase-1 and cleaving GSDMD, accelerating IL-1β/HMGB1 release.	Breast cancer	(78)
Metformin	Caspase-1/GSDMD	Exerting anticancer effects.	Targeting the miR-497/PELP1 axis, activating caspase-1 and cleaving GSDMD	Esophageal squamous cell carcinoma	(108)
	Caspase-1/GSDMD	Suppressing the proliferation and migration of tumor cells.	Upregulating FOXO3 and activating NLRP3/caspase-1/GSDMD pathway, accelerating IL-18/IL-1β release.	Hepatocellular carcinoma	(109)
	Caspase-3/GSDME	Suppressing tumor genesis and progression.	Enhancing AMPK/SIRT1 axis, increasing NK-κB p65, inducing Bax activation and Cyt-C release, leading to caspase-3 activation and GSDME cleavage.	Hepatocellular carcinoma, breast cancer and colon cancer.	(110)
H2	Caspase-1/GSDMD	Protecting healthy cells and suppressing tumor cell growth.	Increasing ROS, activating caspase-1 and cleaving GSDMD, accelerating IL-1β/LDH release.	Endometrial cancer	(111)
Ophiopogonin B	Caspase-1/GSDMD	Restraining tumor progression.	Activating caspase-1 and cleaving GSDMD	Lung cancer	(112)
SDG	Caspase-1/GSDMD	Restraining tumor progression.	Activating caspase-1 and cleaving GSDMD	Colorectal cancer	(93)
Taxo	Caspase-1/GSDMD	Restraining tumor progression.	Activating caspase-1 and cleaving GSDMD	Nasopharyngeal cancer	(113)
Benzimidazoles	NLRP3 /GSDMD	Suppressing tumor growth	Activating NF-κB/NLRP3 and cleaving GSDMD	Glioblastoma multiforme	(114)
ANPS	Caspase-3/GSDME	Protecting healthy cells and suppressing tumor cell growth.	Activating caspase-1 and cleaving GSDME	Lung cancer, cervical cancer and colon cancer	(115)
Galangin	Caspase-3/GSDME	Restraining GBM progression	Inducing caspses-3/GSDME dependent pyroptosis	glioblastoma	(85)
Lobaplatin	Caspase-3/caspase-9/GSDME	Decreasing the viability and proliferation of tumor cells	Inducing ROS generating and JNK phosphorylation, activating caspase-3/9, degrading GSDME and promoting inflammatory cytokine release.	Colorectal cancer	(98)
Cisplatin	Caspase-3/GSDME	Exerting anticancer effects.	Activating caspase-3 and cleaving GSDME.	Lung cancer, oesophageal squamous cell carcinoma,	(116, 117)
	Caspase-3/caspase-9/GSDME	Damaging tumor cells and exerting anticancer effects.	Activating calpain and stimulating CAPN1/CAPN2-BAK/BAX-caspase-9-caspase-3-GSDME signaling axis	Esophageal cancer	(118)
Triptolide	Caspase-3/GSDME	Eliminating tumor cells and exerting anticancer effects.	Suppressing C-myc and HK- II and further activating BAD/BAX-caspase-3 axis, cleaving GSDME and inducing IL-1β/LDH1 release.	Head and neck cancer	(119)
Miltirone	Caspase-3/GSDME	Suppressing proliferation of tumor cells.	Inducing ROS accumulation and inhibiting MEK and ERK1/2 phosphorylation, activating caspase-3, cleaving GSDME and inducing pyroptosis.	Hepatocellular cancer	(60)
Osthole	Caspase-3/GSDME	Exerting anticancer effects.	Activating caspase-3, cleaving GSDME and inducing pyroptosis.	Cervical cancer	(120)
Nitidine chloride	Caspase-3/GSDME	Exerting anticancer effects.	Activating caspase-3, cleaving GSDME and inducing pyroptosis.	Lung cancer	(121)
5-FU	Caspase-3/GSDME	Suppressing viability of tumor cells and exerting anticancer effects.	Activating caspase-3, cleaving GSDME and inducing pyroptosis.	Gastric cancer	(27)
Doxorubicin	Caspase-3/GSDME	Suppressing viability of tumor cells and exerting anticancer effects.	Facilitating intracellular ROS accumulation, inducing JNK phosphorylation, activating caspase-3, cleaving GSDME and inducing pyroptosis.	Breast cancer	(122)
Cirtric acid	Caspase-1/caspase-4/GSDMD	Suppressing proliferation of tumor cells and exerting anticancer effects.	Upregulating TXNIP, activating NLRP3/caspase-1 and degrading GSDMD, releasing IL-18/IL-1β. Activating caspase-4 and degrading GSDMD, inducing pyroptosis.	Ovarian cancer	(123)
α-NETA	caspase-4/GSDMD	Inhibiting the growth and size of tumor.	Activating caspase-4, cleaving GSDMD and inducing pyroptosis.	Epithelial ovarian tumor	(124)
Doxorubicin/epirubicin/daunorubicin/actinomycin D	Caspase-8/GSDMC	Exerting anticancer activity.	Activating caspase-8 cleaving GSDMC and inducing pyroptosis.	Breast cancer	(40)
Thymoquinone	The NLRP3 inflammasome/Caspase-1	Inhibiting proliferation and migration of tumor cells	Inhibiting the NLRP3 inflammasome and suppressing caspase-1 cleavage, inhibiting IL-18/IL-1β release.	Metastatic melanoma	(125)
Simvastatin	The NLRP3 inflammasome/Caspase-1	Exerting anticancer activity.	Activating the NLRP3 inflammasomes, inducing caspase-1 cleavage and promoting IL-18/IL-1β release.	Lung cancer	(126)

### 4.1 Potential cancer-fighting compounds that promote pyroptosis

Among the many molecules involved in the pyroptotic signaling pathway, GSDMD and GSDME have been the two most thoroughly studied with respect to cell membranal hole formation and pyroptosis. They have long been considered as the critical cancer suppressors in various types of cancers, such as renal clear cell carcinoma (127), adrenocortical carcinoma (127), hepatocellular carcinoma (127), primary gastric carcinoma (51), and colorectal carcinoma (128).

#### 4.1.1 Compounds targeting caspase-1/GSDMD

Cisplatin was synthesized as early as 1845 (129) and has been used as traditional antitumor agent for the treatment of a variety of solid tumors, including lung cancer, breast cancer and many others. The widely accepted anti-tumor mechanism of cisplatin is that DNA binds to cisplatin, causing inter- or intra-strand cross-links (51, 130–132),which further leads to deficient DNA template, restriction of DNA synthesis and replication, and DNA damage. Severe DNA damage results in irreversible and serious tumor cell death (131). Recently, Yuan et al. (104) revealed a new mechanism of cisplatin-induced pyroptosis in triple-negative breast cancer. They found that cisplatin could induce MDA-MB-231 cell pyroptosis via upregulating the lnc RNA MEG3 and activating the NLRP3/caspase-1/GSDMD pathway, which would accelerate the inflammatory cytokines (IL-18 or IL-1β) release and effectively treat breast cancer.

Emerging evidence has shown that cucurbitacin B possesses powerful anticancer ability against a variety of cancers by forcing cancer cells into the G2/M phase arrest, resulting in cell death (105). Yang et al. (105) first reported that cucurbitacin B suppressed the progression of non-small cell lung cancer (NSCLC) through pyroptosis *in vivo* and *in vitro*. To be specific, cucurbitacin B can bind to toll-like receptor 4 (TLR4) to stimulate the NLRP3 inflammasome and activate caspase-1, which further leads to the cleavage of the N-terminal of GSDMD to carry out pyroptosis. Consequently, water influx, cell membrane swelling, and the release of numerous pro-inflammatory factors (such as HMGB1, IL-18, and IL-1β) occur. In addition, certain other plant extracts have also been observed to induce pyroptosis and serve as potential cancer suppressors. For instance, anthocyanin was able to restrain oral squamous cell tumor cell viability and suppress proliferation and migration via inducing pyroptosis through the NLRP3/caspase-1/GSDMD pathway (106). In addition, polyphyllin VI (PPVI), the main saponin extracted from *Trillium tschonoskii* Maxim, was reported to markedly inhibit NSCLC proliferation. These *in vitro* experiments clarified that PPVI could activate the NLRP3 inflammasome in a dose-dependent manner in A549 and H1299 NSCLC cell lines. Moreover, the further investigation of the mechanism demonstrated that PPVI could stimulate the NF-κB pathway through increasing ROS levels, further activating the NLRP3/caspase-1/GSDMD signal axis, and inducing pyroptosis in NSCLC (107).

Docosahexaenoic acid (DHA), a rich omega-3 fatty acid, has been proven to exert anticancer effects on breast cancer via apoptosis (133). A recent study revealed a new mechanism by which DHA acts as an anti-breast cancer agent via pyroptosis-programmed cell death. Pizato et al. discovered that accessorial caspase-1 expression, activation of GSDMD, excessive IL-1β release, translocation of HMGB1 to the cytoplasm, membrane hole formation, and other pyroptosis characteristics were observed in breast cancer cells treated with DHA when compared with the untreated group, implying that DHA may be an inducer of pyroptosis in breast cancer cells (78).

Metformin, a classical anti-diabetic drug, has also been explored for many other properties, including cancer resistance (134). What’s more, metformin protects normal tissue from radio- and chemotherapy-induced toxicity while making various malignant cells sensitive to anti-cancer drugs. An increasing number of experiments have demonstrated that the induction of pyroptosis may cause the antitumor properties of metformin. Zhang et al. found that esophageal squamous cell carcinoma (ESCC) cells treated with metformin presented a more serious pyroptototic state, as indicated by the elevated expression of caspase-1 and N-GSDMD when compared to that in the control group (108). In-depth mechanisstic investigation revealed that pyroptosis induced by metformin in ESCC cells is through targeting the miR-497-PELP1 axis. PELP1 is a complex scaffolding oncogene composed of multiple subunits, including proline, glutamic acid and leucine-rich protein 1. In addition, metformin suppressed the proliferation and migration of HCC cells. Further, it inhibited the development of HCC via inducing pyroptosis through upregulation of FOXO3. Excessive FOXO3 activates NLRP3, and consequently, elevated expression of cleaved caspase-1, N-GSDMD, IL-18, and IL-1β etc., will be detected (109).

Interestingly, diatomic hydrogen (H2) demonstrated a dual effect in simutaneously protecting healthy cells and suppressing tumor cell growth. A recent study has shown that H2 pretreatment increased ROS accumulation and pyroptosis-associated proteins, such as NLRP3, caspase-1, GSDMD, and LDH and IL-1β release, in human endometrial cancer (111). In addition to the agents described above, Ophiopogonin B (112), secoisolariciresinol diglucoside (SDG) (93), and taxol (113) were also reported to induce pyroptosis, thus restraining the progression of lung cancer, colorectal cancer, and nasopharyngeal cancer, respectively, through the caspase-1/GSDMD pathway. Excitingly, Ge et al. designed the novel pyroptosis inducer, Cu-TBB, which can specifically target the tumor cells and promote tumor cell pyroptotic death via the caspase-1/GSDMD pathway without damaging normal human cells and organs (41) (**Table 1**).

#### 4.1.2 Compounds targeting caspase-3/GSDME

An increasing number of studies have indicated that the expression level of GSDME determines whether the form of cell death is apoptosis or pyroptosis in caspase-3-activated cells. Cells with high expression of GSDME go through pyroptosis after “apoptosis stimulation,” such as that induced by chemotherapy. Oppositely, cells expressing low levels of GSDME undergo secondary necrosis following apoptosis (15, 50). The majority of cancer tissues express GSDME at low levels due to the increased methylation of promoter CpGs compared to that in healthy samples (135, 136). Decitabine could reverse the low expression of GSDME in cancer cells and enhance the sensitivity of cancer cells to chemotherapy medicines (51, 52, 137). In addition, Yu et al. (98) discovered that lobaplatindecreases the viability and proliferation of HCT116 and HT-29 cells, two types of CRC cell lines, in a dosage-dependent manner. The cells treated with lobaplatin performed the classic pyroptotic features of cellular swelling and membrane pores. An in-depth mechanistic exploration revealed that the addition of lobaplatin induced ROS production and JNK phosphorylation, promoting Bax mitochondrial translocation and accelerating cytochrome C transport to the cytosol, leading to caspase-3/9 activation, GSDME cleavage, and, ultimately, pyroptosis (**Table 1**).

Cisplatin exerts anti-cancer effects on various tumors through multiple mechanisms. Zhang et al. (116) discovered that caspase-3 activation and GSDME-NT generation increased significantly after cisplatin treatment of A549 cells, a kind of lung cancer cell lines. The caspase-3 inhibitor Ac-DEVE-CHO and GSDME knockdown markedly inhibited cisplatin-induced GSDME cleavage and simultaneously suppressed pyroptosis. These results suggest that cisplatin induces pyroptosis via the caspase-3/GSDME pathway in A549 cells, which may provide a potential therapeutic target for the clinical treatment of lung cancer. Additionally, Wu et al. reported that the combination of cisplatin and BI2536, a PLK1 inhibitor,triggered pyroptosis in ESCC cells via the caspase-3/GSDME signaling pathway (117). Furthermore, Li et al. uncovered an innovative mechanism by which cisplatin triggers pyroptosis through the activation of the CAPN1/CAPN2-BAK/BAX-caspase-9-caspase-3-GSDME axis in esophageal cancer cells (118). A previous study revealed that GSDME was abundantly expressed in HeLa cervical cancer cells, SH-SY5Y neuroblastoma cells, and MeWo skin melanoma cells. GSDME-positive SH-SY5Y cells show classical pyroptotic characteristics through caspase-3 cleavage of GSDME following treatment with the chemotherapeutic drugs etoposide topotecan, cisplatin, or irinotecan (138).

Triptolide, an active diterpenoid epoxide ingredient isolated from the herbal medicine *Tripterygium wilfordii*, has been shown to exhibit anti-immunosuppressive, anti-inflammatory, and anti-cancer activities via apoptotic signaling and various other mechanisms (139, 140). Cai et al. have demonstrated that triptolide treatment triggers GSDME-mediated pyroptotic death in head and neck cancer cells (119). The expression level of C-myc and mitochondrial hexokinase II was suppressed in cancer cells treated with triptolide, which would accelerate mitochondrial translocation of BAD/BAX, leading to caspase-3 activation, GSDME cleavage, IL-1β, and LDH1 release, and pyroptosis ultimately. In addition, the authors discovered that triptolide inhibited the NRF2/ALC7A11 axis and induced ROS accumulation regardless of GSDME content. Thus, triptolide may be a promising drug for treating head and neck malignancies (119).

Miltirone, an active diterpene quinine constituent extracted from the Chinese herb *Salvia miltiorrhiza* Bunge, has also been discovered to play an anti-tumor role in various tumors (141, 142). For instance, Wang et al. demonstrated that miltirone causes mitochondrial dysfunction, p53- and ROS- dependent apoptosis in colon tumor cells (143). In addition, Wu et al. have shown that miltirone induces collateral sensitivity in multidrug-resistant P-glycoprotein-overexpressing lymphoblastic leukemia cells. In addition, miltirone arrests G2/M and induces apoptosis through the ROS-mediated breakdown of MMP and DNA damage (144). Zhang et al. identified that miltirone suppresses the proliferation of HepG2 and Hepa1-6 cells in a time- and dose-dependent manner. Further investigation suggested that HepG2 and Hepa1-6 cells treated with miltirone exhibited membrane pores, cell swelling and other pyroptotic characteristics via the caspase-3/GSDME signaling pathway. In addition, miltirone effectively induced intracellular ROS accumulation and inhibited MEK and ERK1/2 phosphorylation to induce pyroptosis in HCC cells (60). Other compounds extracted from traditional Chinese herbs, such as osthole, isolated from the *Cnidium monnieri (L.) Cusson.*, and nitidine chloride, isolated from *Zanthoxylum nitidum (Roxb.) DC*, have been shown to induce caspase-3/GSDME-mediated pyroptosis and are beneficial for the prevention and treatment of cervical and lung cancers, respectively (120, 121).

Metformin has been demonstrated to suppress various tumorigenesis and tumor progression through numerous mechanisms and can inhibit carcinoma development by inducing pyroptosis via different signaling pathways. Zheng et al. identified that metformin exerted its antitumor effect through enhancing the AMPK/SIRT1 axis, subsequently increasing expression of NK-κB p65 to induce Bax activation and Cyt-C release, leading to caspase-3-dependent cleavage of GSDME and ultimately resulting in pyroptosis in HCC cells, breast cancer cells, and colon cancer cells (110). Another classic antitumor medicine, 5-FU, has been reported to suppress cell viability, promote LDH release, induce membrane bubble formation, and cause pyroptotic cell death via the caspase-3/GSDME pathway in SGC-7901 and MKN-45 gastric cancer cell lines (27). Furthermore, GSDME knockout switched pyroptosis induced by 5-FU into apoptosis in gastric cancer cells. Doxorubicin, a representative anthracyline, is a prominent anticancer drug used in multiple cancer chemotherapies. However, its administration is often accompanied by irreversible and continuous cardiotoxic side effects. Zhang et al. showed that breast cancer cells (MDA-MB-231 and T47D) treated with doxorubicin showed decreased cell viability and typical pyroptotic morphology. Mechanism experiments showed that doxorubicin administration facilitated intracellular ROS accumulation, induced JNK phosphorylation, caspase-3 activation, and GSDME cleavage (122). Yu et al. reported that eEF-2K is activated by doxorubicin in melanoma cells, suppressing pyroptosis and promoting autophagic responses. Silencing of eEF-2K blunts autography and expedites doxorubicin-induced pyroptosis via caspase-3/GSDME (145).

To minimize the toxic side effects of chemotherapy drugs on normal human cells and tissues, Wang et al. engineered an acid-activatable nano-photosensitizer (ANPS) with different pH responses using ultra-pH-sensitive nanotechnology (115). Researchers have verified that ANPS efficiently elicits caspase-3/GSDME-mediated pyroptosis in cancer cells by specifically activating phospholipase C and triggering subsequent signal transduction via lipid peroxidation, a process launched by nano-photosensitizer-mediated oxidative stress in early endosomes. In late endosomes and lysosomes, the pyroptosis-promoting ability was dramatically decreased. Thus, the newly-designed pyroptosis nano-tuner ANPS, has significant therapeutic effects on human tumors, concurrently accompanied by minimal toxic side effects on normal human cells and organs (115) (**Table 1**).

#### 4.1.3 Compounds targeting caspase-4/GSDMD

Citric acid (CA) isan organic acid that broadly serves as an antioxidant in cosmetic and food additives (146). It is also a key metabolin during the tricarboxylic acid cycle, and plays an essential role in macromolecule synthesis, energy metabolism, and maintenance of cellular redox balance. Zhao et al. (123) reported that CA suppresses the proliferation of A2780 and SKOV3 ovarian cancer cells in a dose-dependent manner. The morphology of cells treated with CA exhibits representative pyroptosis characteristics, namely swelling and rupture of the cell membrane and cytoplasmic leakage (123). Compared with that in the untreated cell group, the expression of TXNIP and caspase-1 was significantly elevated in the cell group treated with CA. In addition, the content of NLRP3, caspase-1, GSDMD, IL-18, and IL-1β was markedly up-regulated in cells treated with CA, implying that CA accelerates pyroptosis of ovarian cancer cells via the TXNIP/NLRP3/caspase-1/GSDMD axis-mediated canonical and caspase-4/GSDMD-mediated non-canonical pyroptosis pathways (123). Additionally, α-NETA couldincrease the abundance of pyroptosis-related proteins in epithelial cancer cells and inhibit the growth and size of epithelial ovarian tumor in mice via the caspase-4/GSDMD pathway (124).

#### 4.1.4 Compounds targeting other pyroptosis signaling pathways

The crucial immune-checkpoint programmed death ligand 1 (PD-L1) inhibits anticancer immunity by interacting with the programmed cell death protein 1 (PD-1) (147). Hou et al. identified that PD-L1 could switch apoptosis induced by TNF-α into pyroptosis in cancer cells (40). Virtually all chemotherapy medicines can induce PD-L1 translocation and upregulation of GSDMC, whereas only the antibiotics such as doxorubicin, epirubicin, daunorubicin, and actinomycin D can activate caspase-8, induce GSDMC cleavage, and induce pyroptosis in MDA-MB-231 breast cancer cells. However, this phenomenon can be abolished by the PD-L1-NLS, Stat3-Y705F mutation, and caspase-8 knockout (40). Thus, these drugs exhibit anti-tumor activity, probably through strong inflammatory responses in PD-L1^+^ or GSDMC^+^ breast cancers.

Inflammasomes play complex roles in tumorigenesis and cancer development. For example, the NLRP3 inflammasome can serve as an antitumor compound in colitis-associated CRC. However it can also promote cancer progression in skin and gastric cancers (148, 149). As shown in **Table 1**, Ahmad et al. discovered that thymoquinone, the major component of N*igella sativa*, presented an inhibitory effect on metastatic melanoma cell lines. Simultaneously, the thymoquinone-mediated inhibition process of migration was accompanied by NLRP3 decrease, caspase-1 cleavage inhibition, and IL-1β/IL-18 release inhibition (125). Feng et al. demonstrated that the NLRP3 inflammasome accelerates resistance to 5-FU in OSCC *in vivo* and *in vitro*. Inhibiting the ROS/NLRP3/IL-1β signaling pathway may be conducive to 5-FU-based chemotherapy of OSCC (150). Furthermore, the antitumor effects of inflammasomes have been confirmed by multiple studies. Simvastatin, a classical anti-hyperlipidemic drug, was reported to induce pyroptosis by activating the NLRP3 inflammasomes caspase-1-IL-18 and IL-1β axes in H1299 and A549 lung cancer cell lines without causing poisonousness tohealthy lung cells (126). In summary, whether activating or inhibiting inflammasomes is beneficial for clinical treatment is in a tumor -specific manner.

### 4.2 The adverse effects of pyroptosis and compounds inhibiting pyroptosis

In general, the tumor microenvironment is formed by a chronic inflammatory course in which polarized macrophages and stromal components accelerate tumor development. The hypoxic environment near the necrotic area of solid tumors is conducive to the accumulation of macrophages and the secretion of TNF-α, which is vital for tumor necrosis (151–153). Additionally, the hypoxic environment elevates the content of GSDMC that is cleaved via TNF-α-activated caspase-8, contributing to pyroptosis. It’s worth noting that pyroptosis plays a dual role in regulating the tumor’s surroundings. Chronic tumor pyroptosis inhibits antitumor immunity and fuels tumor development (40). In contrast, the acute inflammatory effects induced by pyroptosis in the tumor microenvironment promote the anti-tumor immune response and restrict tumor growth (40, 154–156). Therefore, pro-pyroptotic compounds that induce an acute inflammatory immune response in the tumor microenvironment deserve in-depth exploration in the clinic.

However, scientists reported that GSDME was highly expressed in only 1/10 of tumor cells, but 3/5 of normal human cells exhibited rich expression of GSDME (4). Moreover, researchers have discovered that GSDME-induced pyroptosis is probably the mechanism underlying the toxic side effects caused by chemotherapy drugs (15). For instance, Shen et al. clarified that the classical chemotherapeutic medicines, cisplatin and doxorubicin, trigger GSDME-induced pyroptosis in human tubular epithelial cell lines via the ROS/JNK/caspase-3/GSDME signaling pathway in a concentration- and time-dependent manner (157). Additionally, mice treated with Ac-DMLD-CMK, a polypeptide that inhibits caspase-3 and GSMDE activation, exhibited alleviated deterioration in kidney function and renal tubular epithelial cell damage, as well as decreased inflammatory factor secretion (157). The above findings indicate that caspase-3/DSDME-mediated pyroptosis causes chemotherapy-induced nephrotoxicity. Zhang et al. reported that cardiotoxicity induced by doxorubicin during breast cancer treatment was mediated by caspase-3/GSDME-caused pyroptosis (122). Furthermore, Gao et al. showed that higher GSDMD expression was closely related to adverse outcomes, including larger tumor volume, more advanced tumor-node metastasis periods, and a poorer prognosis in lung adenocarcinoma (68). GSDMD depletion suppresses tumor proliferation by accelerating apoptosis and restraining the EGFR/Akt signaling pathway in NSCLC (68).

Interestingly, Molina-Crespo Á et al. developed a novel targeted nanomedicine that was able to send the specific anti-GSDMB antibody into the HER2-positive breast cancer cells *in vivo* and *in vitro*, based on hyaluronic acid-biocompatible nanocapsules (158). This newly developed nanomedicine specifically and significantly suppressed GSDMB function and inhibited cancer cell migration and metastasis. In addition, resistance to chemotherapy is simultaneously inhibited (158). Disulfiram, a drug used to treat alcohol addiction, suppresses canonical and non-canonical inflammasome pyroptosis signaling pathways to curb cell death. In addition, disulfiram prevents hole formation in the cell membrane via covalently modifying Cys191/Cys192 in GSDMD and prevents the the liposome leakage in cleaved GSDMD (159). Additionally, Humphries et al. discovered that exogenous dimethyl fumarate or endogenous fumarate reacts with GSDMD to produce 2-(succinyl)-cysteine at Cys191 and Cys192 in human and mouse GSDMD, respectively, which prevents the interaction between GSDMD and caspases, suppressing its processing and ability to induce pyroptotic cell death. These findings highlight the potential of dimethyl fumarate in treating inflammatory diseases, including familial Mediterranean fever, multiple sclerosis, demyelination, and experimental autoimmune encephalitis (160).

Presently, our understanding of the specific roles and effects of pyroptosis in various tumors and the detailed mechanisms by which these factors affect the tumor cells has not been sufficiently elucidated. Furthermore, there are conflicting results on the relationship between tumors and pyroptosis due to the complexity of tumor heterogeneity and the immune microenvironment. Therefore, any pyroptosis agonists or inhibitors developed will need long-term experimental clinical research to assess their specific clinical outcome fully.

## 5 Summary and prospect

Pyroptosis, a type of lytic and necrotic-regulated cell death executed by the gasdermin family, is characterized by DNA fragmentation, chromatin condensation, hole formation in the cell plasma membrane, cell bulking, content release, and cell rupture (44, 161). As an inflammatory death pattern, pyroptosis plays an essential role in tumor limitation via enhancing the sensitivity of various tumor cells to chemotherapy drugs and generating many neo-antigens that consequently galvanize systemic immunity (15, 50, 162). However, the tumor microenvironment created by chronic tumor pyroptosis fuels tumor development. Scientists have stated that GSDME is expressed at low levels in the majority of cancer cells because of promoter hypermethylation in tumors. In contrast, it is highly expressed in most normal healthy cells (15, 50). Thus, the caspase-3-mediated pyroptosis induced by chemotherapy drugs in healthy cells rich in GSDME might be the reason for the side effects and toxicity of chemotherapy drugs.

While significant progress has been achieved in investigating the pathological significance and mechanisms of pyroptosis in various tumors, several crucial and indispensable inquiries still need to be addressed and resolved for the future implementation of therapeutic strategies targeting pyroptosis. Firstly, given the complex functions and dual effects of pyroptosis in tumors, attaining optimal therapeutic outcomes by accurately regulating the disease at various stages remains challenging. Secondly, further studies are required to explore effective measures to prevent the chronic inflammatory responses during the induction of cancer cell pyroptosis. Thirdly, there is a need for further investigation into how to induce tumor cells that have developed resistance to apoptosis to transition toward pyroptosis, with clinical data necessary to verify its feasibility. Addressing the scientific challenges highlighted in this review will enhance our understanding of the crucial involvement of pyroptosis in various tumor types, thereby establishing a solid scientific foundation for utilizing pyroptosis as a target for the prevention, treatment, diagnosis, and prognosis of multiple tumors.

## Author contributions

H-MY: Software, Writing – original draft, Writing – review and editing. LW: Project administration, Writing – original draft, Writing – review and editing. HM: Writing – review and editing. CH: Writing – review and editing. G-YF: Supervision, Writing – review and editing. JL: Conceptualization, Funding acquisition, Supervision, Writing – review and editing.

## Glossary

**Table:**

AIM2	absent in melanoma 2
ASC	apoptosis-associated speck-like protein containing a caspase-recruitment domain
CA	Citric acid
CARD	caspase activation and recruitment domain
CRS	cytokine release syndrome
CTLs	cytotoxic T lymphocytes
DAMP	danger-associated molecular patterns
DHA	Docosahexaenoic acid
ERK1/2	extracellular regulated protein kinase 1/2
ESCC	esophageal squamous cell carcinoma
GSDMD	gasdermin D
GZMA	granzyme A
GZMB	granzyme B
H2	diatomic hydrogen
HCC	hepatocellular carcinoma
hUCMSCs	human umbilical cord mesenchymal stem cells
HMGB1	high mobility group box1
IL	Interleukin
LPS	lipopolysaccharide
MEK	mitogen-activated and extracellular signal-regulated kinase
MM	malignant mesothelioma
NLRs	the nucleotide-binding oligomerization domain-like receptors
NKs	natural killer cells
NSCLC	non-small cell lung cancer
NSCLCs	non-small cell lung cancer cells
PAMP	pathogen-associated molecular patterns
PCD	programmed cell death
PD-1	Programmed cell death protein 1
PD-L1	Programmed cell death ligand 1
PPVI	polyphyllin VI
PRRs	pattern recognition receptors
PYD	pyrin domain
SDG	Secoisolariciresinol diglucoside
TLR4	Toll-like receptor 4
